# 422. Impact of Respiratory Virus Co-Infection in Pediatric COVID-19

**DOI:** 10.1093/ofid/ofad500.492

**Published:** 2023-11-27

**Authors:** Manvita Mareboina, Katrina Bakhl, James Fisher, Simrat Jassal, Jessica E Ericson, Patrick Gavigan

**Affiliations:** Pennsylvania State College of Medicine, State College, Pennsylvania; Pennsylvania State University College of Medicine, State College, Pennsylvania; Penn State Milton S Hershey Medical Center, Hershey, Pennsylvania; Pennsylvania State University College of Medicine, State College, Pennsylvania; Penn State College of Medicine, Hershey, Pennsylvania; Department of Pediatrics, Penn State College of Medicine, Hershey, Pennsylvania

## Abstract

**Background:**

Severe acute respiratory syndrome coronavirus 2 (SARS-CoV-2) is a viral infection that emerged in December 2019, leading to the COVID-19 pandemic. Despite significant research, there are still significant knowledge gaps, particularly with regards to COVID-19 in children and the impact of respiratory virus co-infection.

**Methods:**

A retrospective chart review was conducted at Penn State Children’s Hospital including children who tested positive for SARS-CoV-2 by multiplex polymerase chain reaction from nasopharyngeal swabs between March 2020 and June 2022. Demographics, symptoms, clinical features, and treatments of patients who tested positive for SARS-CoV-2 alone were compared to those who had co-detection of another respiratory virus.

**Results:**

A total of 464 episodes of SARS-CoV-2 detection were included, with 123 (26.5%) involving of co-detection of another respiratory virus. Patients with viral co-detection more commonly presented with upper respiratory tract infection (URI) symptoms and, cough, and were more likely to require any supplemental oxygen therapy. However, there were no differences in rates of mechanical ventilation, intensive care unit admission, mortality, or receipt of SARS-CoV-2 specific antivirals [Table 1]. Co-detections occurred throughout the study period [Figure 1]. Of the 123 episodes of viral co-detection, 99 (80.5%) had one other virus detected. The most commonly co-detected virus was rhino/enterovirus, which was found in 100 (81.3%) of the co-detection episodes.

Outcomes of SARS-CoV-2 and Viral Co-Detection

**Figure:**
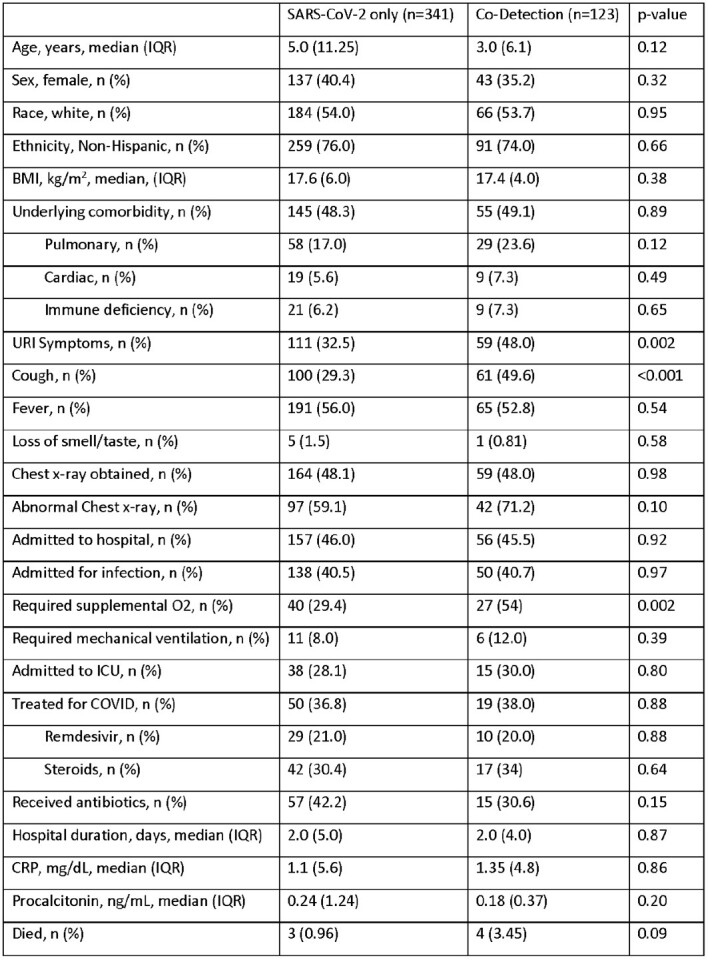

Numbers of SARS-CoV-2 and Viral Co-Detections

**Figure:**
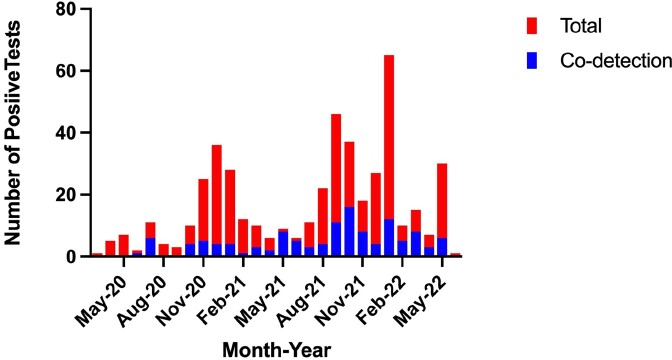

**Conclusion:**

This study provides insight into the impact of respiratory virus co-infections in pediatric COVID-19. Co-infection was more often associated with URI symptoms, but otherwise appeared clinically similar to SARS-CoV-2 mono-detection. Additionally, while children with co-infection more often needed any supplemental oxygen, there were no differences with regard to other markers of clinical severity. As SARS-CoV-2 continues to circulate with other respiratory viruses, ongoing studies will be needed to determine if alternative treatment strategies are needed.

**Disclosures:**

**Jessica E. Ericson, MD, MPH**, Abbvie: Advisor/Consultant

